# The Prevalence and Magnitude of Impaired Cutaneous Sensation across the Hand in the Chronic Period Post-Stroke

**DOI:** 10.1371/journal.pone.0104153

**Published:** 2014-08-14

**Authors:** Jocelyn L. Bowden, Gaven G. Lin, Penelope A. McNulty

**Affiliations:** 1 Neuroscience Research Australia, Sydney, New South Wales, Australia; 2 University of New South Wales, Sydney, New South Wales, Australia; University of Ottawa, Canada

## Abstract

Sensation is commonly impaired immediately post-stroke but little is known about the long-term changes in cutaneous sensation that have the capacity to adversely impact independence and motor-function. We investigated cutaneous sensory thresholds across the hand in the chronic post-stroke period. Cutaneous sensation was assessed in 42 community-dwelling stroke patients and compared to 36 healthy subjects. Sensation was tested with calibrated monofilaments at 6 sites on the hand that covered the median, ulnar and radial innervation territories and included both glabrous (hairless) and hairy skin. The motor-function of stroke patients was assessed with the Wolf Motor Function Test and the upper-limb motor Fugl-Meyer Assessment. Impaired cutaneous sensation was defined as monofilament thresholds >3 SD above the mean of healthy subjects and good sensation was ≤3 SD. Cutaneous sensation was impaired for 33% of patients and was 40–84% worse on the more-affected side compared to healthy subjects depending on the site (p<0.05). When the stroke patient data were pooled cutaneous sensation fell within the healthy range, although ∼1/3 of patients were classified with impaired sensation. Classification by motor-function revealed low levels of impaired sensation. The magnitude of sensory loss was only apparent when the sensory-function of stroke patients was classified as good or impaired. Sensation was most impaired on the dorsum of the hand where age-related changes in monofilament thresholds are minimal in healthy subjects. Although patients with both high and low motor-function had poor cutaneous sensation, overall patients with low motor-function had poorer cutaneous sensation than those with higher motor-function, and relationships were found between motor impairments and sensation at the fingertip and palm. These results emphasize the importance of identifying the presence and magnitude of cutaneous sensory impairments in the chronic period after stroke.

## Introduction

Sensory receptors in the skin of the hand provide information about the external environment that is important for fine motor control of the hand [1]. Human motor control is predicated on sensorimotor integration. The interaction between cutaneous receptors and the motor system is evident from changes to ongoing muscle activity that is temporally coupled to sensory signals reaching the spinal cord. These cutaneomuscular spinal reflexes can be demonstrated by the activation of populations of cutaneous receptors [2], single receptors [3], [4], and between single receptors and single motor units [5]. Without appropriate sensory information the motor system may be more severely disabled than after a purely motor stroke. Stroke survivors with impaired sensation report reduced dexterity or clumsiness sufficient to interfere with personal safety, self-care and independence in activities of daily living [6]. Yet the magnitude of sensory impairment in community dwelling stroke patients has not been quantified and the relationship between post-stroke sensory impairments and motor performance is poorly understood, especially once patients have returned to the community.

Sensation is commonly impaired immediately after stroke. In the acute and early sub-acute period conscious touch perception is reduced by up to 85% on the more-affected side [7]–[10], and 25% on the less-affected side [7], [8], [11]. Changes in cutaneous sensation may occur in isolation or with altered proprioception [7] or global sensory and motor dysfunction [12]. Impairments are defined as “problems in body function or structure that result in a significant deviation or loss” [13]. Body functions and structure in the periphery, such as somatosensory receptors and their afferent pathways are not directly damaged by stroke, rather the nature of sensory loss is related to lesion location [14]. However, secondary deficits may occur subsequently in healthy neurons remote but functionally coupled to the focal lesion through diaschisis [15] and cortical reorganization [16]. The prevalence of long-term sensory deficits >6 months post-stroke has been estimated at 37%, although the impairments were not quantified or described [17], [18].

Reduced sensation is associated with poor recovery of physiological function related to movement, referred to here as motor-function. These changes in motor-function may include slower improvements in the hemiplegic arm, delayed movement onset and impaired reach trajectories [19]–[23]. Consistent relationships between reduced sensation in different modalities (such as cutaneous or proprioceptive sensation) and motor-function have not been found [23]–[25], but long-term changes in descending drive and spinal reflexes [26], [27] are thought to trigger secondary adaptive changes to the peripheral neuromusculature [28]. This may be exacerbated in patients with low motor-function in whom decreased movement contributes to reduced sensory signaling, although this relationship has not been studied.

Changes in cutaneous sensation after stroke may be compounded by those that occur as a function of age. While stroke *per se* is not age-dependent, the majority of strokes occur after 60 years of age [29], [30], a period of accelerating decline in both sensory and motor-function [31], [32]. We previously demonstrated that age-related declines in cutaneous sensation of the hand varied between the fingertips, palm and dorsal regions [31]. The association between changes in motor control and cutaneous sensation with ageing is not strong [33], and has not been tested after stroke.

There is no widely-accepted or standardized test of sensory impairments after stroke [34]. Impairments in cutaneous sensation are usually assessed at the index fingertip [9], [35], or clinically by descriptors such as ‘present’, ‘absent’ or ‘impaired’ [8], [18], [36], [37]. The Fugl-Meyer Assessment (FMA) is a common measure of post-stroke motor performance. It assesses both impairments and reflexes, and how well patients can execute simple motor tasks. In addition to the widely-used motor subscale, the FMA includes a sensory subscale that despite good inter-rater reliability [37], may have significant ceiling effects [38]. These tools may identify the presence but not the magnitude of sensory impairments across the hand after stroke. Other tools routinely used to quantify cutaneous sensation in healthy ageing population include the grating orientation test, gap detection, and pattern discrimination tasks [39]–[41]. Many of these assessments were not appropriate for this study due to the physical and cognitive limitations of the stroke cohort. Monofilaments were chosen as they apply a known force to a small area of skin, allowing quantification of fine touch across the hand. Thresholds of ≥0.6 g have previously been classified as *diminished protective sensation* that significantly impacts fine motor control of the hand [42]. Further, monofilaments can be used to assess sensation in patients with low motor-function and aphasia.

The aim of this study was to quantify the magnitude of persistent cutaneous sensory impairments in community-dwelling stroke patients presenting with an upper-limb motor disability. Motor dysfunction continues to be studied in detail after stroke, but sensory dysfunction much less so. In order to facilitate rehabilitation of sensorimotor integration after stroke, it is necessary to understand both sensory and motor dysfunction, and to ensure analyses are based on quantifiable classification systems. This is the first study to quantify cutaneous thresholds for stroke survivors in the chronic phase; the first to quantify sensation at multiple sites on the hand; to classify impairments for different regions of the hand in comparison to healthy age-appropriate subjects; and to compare the relationship between sensory declines at these sites in patients with both high and low motor-function. We hypothesized that patients with low motor-function would have greater impairments in cutaneous sensation across the hand than those with high motor-function, and that sensory and motor impairment would be positively correlated.

## Methods

### Subjects

Cutaneous sensory thresholds and upper-limb motor-function were investigated in 42 consecutively recruited stroke patients aged 18–80 years (60.1±14.0 years [mean, SD]; 28 males, 14 females) and 3–264 months post-stroke (28.2±47.3 months). All patients had hemiparesis including an upper-limb involvement following a unilateral stroke in the territory of the middle cerebral artery (see Table 1), and were community-dwelling after discharge from in- and out-patient rehabilitation. Patients’ cutaneous thresholds were compared to those of 36 neurologically healthy subjects selected from an historical cohort of community volunteers [31]. Healthy subjects were matched to the patients for age and sex (60.4±15.0 years; 22 males, 14 females). All participants were screened for overt sensory or motor impairments associated with diabetes, peripheral neuropathies, or other non-stroke conditions. Patients with expressive aphasia were included in the study. The motor-function (see *Motor and sensory classification of the patient group*) of 14 stroke patients included in this novel analysis has been reported previously [43], [44].

**Table 1:** Demographic data and motor scores of stroke patients classified by more-affected side motor-ability and sensory function.

	Motor function		Sensory function	
	High motor-functiongroup	Low motor-functiongroup	Good sensationgroup	Poor sensationgroup
n	24	18	28	14
Age (mean, SD)	62.7±10.2	56.7±17.6	63.2±11.6	53.9±16.5
Sex (male:female)	17∶7	11∶7	18∶10	10∶4
Months post-stroke (mean, SD)	21.5±36.5	37.2±58.7	31.8±56.8	21.2±16.5
More-affected side (right:left)	11∶13	7∶11	14∶14	10∶4
Dominant side affected (n)	10	8	13	11
Stroke type Ischaemic:hemorrhagic	19∶5	10∶8	20∶8	9∶5
Wolf Motor Function Test (s)	4 [3]–[12]	97 [74–113]***	12.1 [3.8–70.2 ]	82 [12–113]
Fugl-Meyer Assessment	57±1.4	16±3.2***	55 [27–61]	17 [5]–[55]*

Motor-function scores were different between groups (***p<0.001). Fugl-Meyer scores were higher for patients with poor sensation (*p<0.05). Wolf Motor-Function Test data are the mean times for the 15 timed tasks with a maximum time of 120 s per task; maximum Fugl-Meyer score is 66. Data are presented as mean ± SEM or median [IQR].

### Experimental procedures

Cognitive competency was assessed as a Mini-Mental State Examination score ≥24 or verified by treating physicians for patients with expressive aphasia. All participants gave written, informed consent. The study was approved by the Human Research Ethics Committees of St Vincent’s Hospital and the University of New South Wales, Sydney, Australia and conducted in accordance with the Declaration of Helsinki.

After all tests were explained and demonstrated, the less-affected side of patients, or the dominant side of healthy subjects, was tested first to allow familiarity with the procedures on the better performing side. Healthy subjects nominated their dominant hand. Sensory assessments were performed in the order described below by the first 2 authors following a standardized protocol.

#### Cutaneous sensation

**Figure 1:**
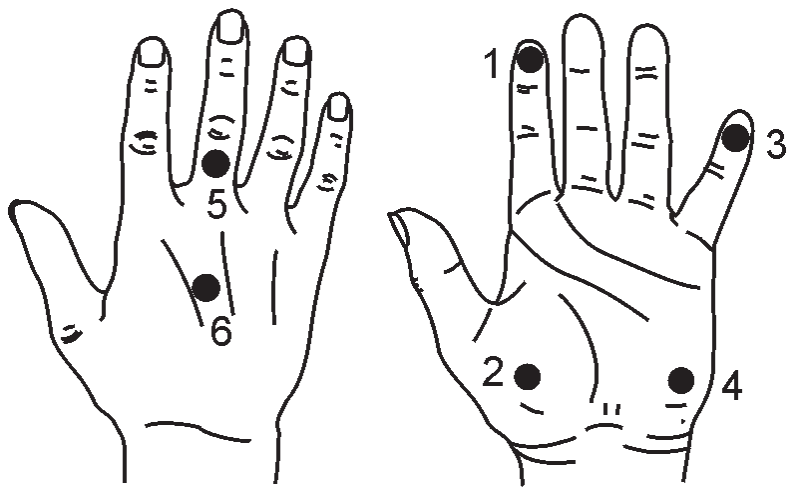
Cutaneous sensation testing sites. Sites 1–4 reflect glabrous skin and sites 5–6 hairy skin. Sites 1–2 have median nerve innervation, 3–4 ulnar nerve, and 5–6 radial nerve.

Cutaneous thresholds were measured using calibrated von Frey monofilaments (North Coast Medical, USA) at 6 sites (Fig. 1) according to established protocols [31]. Assessments were performed in a climate controlled laboratory to control for temperature and humidity [45]. Subjects sat with the test hand resting on a table and masked from view. Order and anticipation bias were avoided by randomly altering the location of, and length of time between stimulus presentations. Stimuli were first applied to one of the four sites on the palmar surface. When testing of these sites was completed, stimuli were then randomly applied to one of the two sites on the dorsum. The monofilament size was decreased until the stimuli were no longer perceived, and subsequently increased to confirm the threshold. Stroke patients acknowledged a stimulus either by verbal confirmation of the site or by pointing with the opposite hand. Healthy subjects identified the stimulus according to a numbered picture (Fig. 1). The lowest perceived filament was recorded as the threshold (g). Care was taken to ensure a single stimulus was applied orthogonal to the skin surface at each presentation. If the filament slipped or made multiple contacts on the skin, the stimulus was repeated as part of the random sequence. Bilateral sensory assessments were performed during the same 30 minute session.

#### Other assessments after stroke

Post-stroke motor-function was assessed on the more-affected side using two common tests; the Wolf Motor Function Test timed-tasks (WMFT) [46] and the upper-limb motor FMA [47]. Wolf-Motor Function Test scores are calculated as the mean of the summed times for 15 timed tasks that simulate activities of daily living. They are graded from simple gross movements such as lifting the arm, to finer tasks of finger dexterity required to pick up small objects. The FMA assesses both individual and synergistic movements of the shoulder, wrist and hand. Faster times and a higher score respectively indicate better motor-function and less motor impairment. Use of the more-affected side was quantified in a subset of patients (n = 30) using the Motor Activity Log Quality of Movement Scale (MALQOM) [48]. The MALQOM is a self-rated questionnaire consisting of 30 activities of daily living. It is scored on a 6-point Scale with 0 representing an inability to complete the task and 5 representing the same ability as before the stroke.

#### Motor and sensory classification of the patient group

The patients were classified by motor-function (low and high) and cutaneous sensory function (poor and good). Classification criteria were determined *a priori.* Low motor-function was based on previous rehabilitation studies [49] and defined as active wrist and finger extension on the more-affected side <20°, and high motor-function as extension ≥20°. As there are no accepted classification systems for cutaneous sensation, poor sensation or the “*zone of impairment*”, was defined as monofilament thresholds >3 SD from the mean of healthy age-appropriate subjects at ≥2 sites [31]. Each patient was then compared to the mean threshold for the age-appropriate subjects, for each site. The mean thresholds were >0.6 g for fingertip sites, >1 g for palmar sites and >0.3 g for dorsum sites. Fingertips thresholds of ≥0.6 g have previously been suggested to represent a loss of protective sensation [42].

#### Data and Statistical Analysis

Monofilament thresholds were not different between hands for healthy subjects when tested with Mann-Whitney Rank Sum Tests. Therefore data are reported for both hands combined. Likewise, because there were no differences for cutaneous sensation between the fingertips (sites 1, 3), palm (sites 2, 4), or the dorsum (sites 5, 6) data for each pair were averaged and hereafter referred to as regions for all participants.

Cutaneous sensory thresholds were assessed separately for the fingertip, palm and dorsum using a Kruskal-Wallis one-way ANOVA on ranks with Tukey *post hoc* analyses. Side tested was the main factor (more-affected, less-affected, healthy). The data were then re-analyzed using Kruskal-Wallis one-way ANOVAs with motor or sensory classification incorporated into the side-tested. Patients with monofilament thresholds greater than the maximal measureable threshold were coded 301 g for analysis. Spearman rank order correlations were calculated using raw scores to examine relationships between cutaneous sensation: i) at the fingertip and palm; ii) at each region against WMFT and FMA scores; and iii) at each region against time-since-stroke and age. Data are reported as median and interquartile range [IQR] unless otherwise noted. Differences were considered significant when *p*<0.05.

## Results

### Cutaneous sensation

Increased monofilament thresholds reflect a decrease in cutaneous sensation. According to the criteria outlined in the Methods, 14 patients had impaired sensation (33%) of whom (6 with low motor-function, 4 with high motor-function) had impaired thresholds on the more-affected side only. Another single patient with high motor-function had a bilateral impairment. Two patients with low motor-function could not perceive the largest monofilament (300 g) at any region on the more-affected side, and another also with low motor-function on the palm and fingers.

#### Unclassified stroke patients

**Figure 2:**
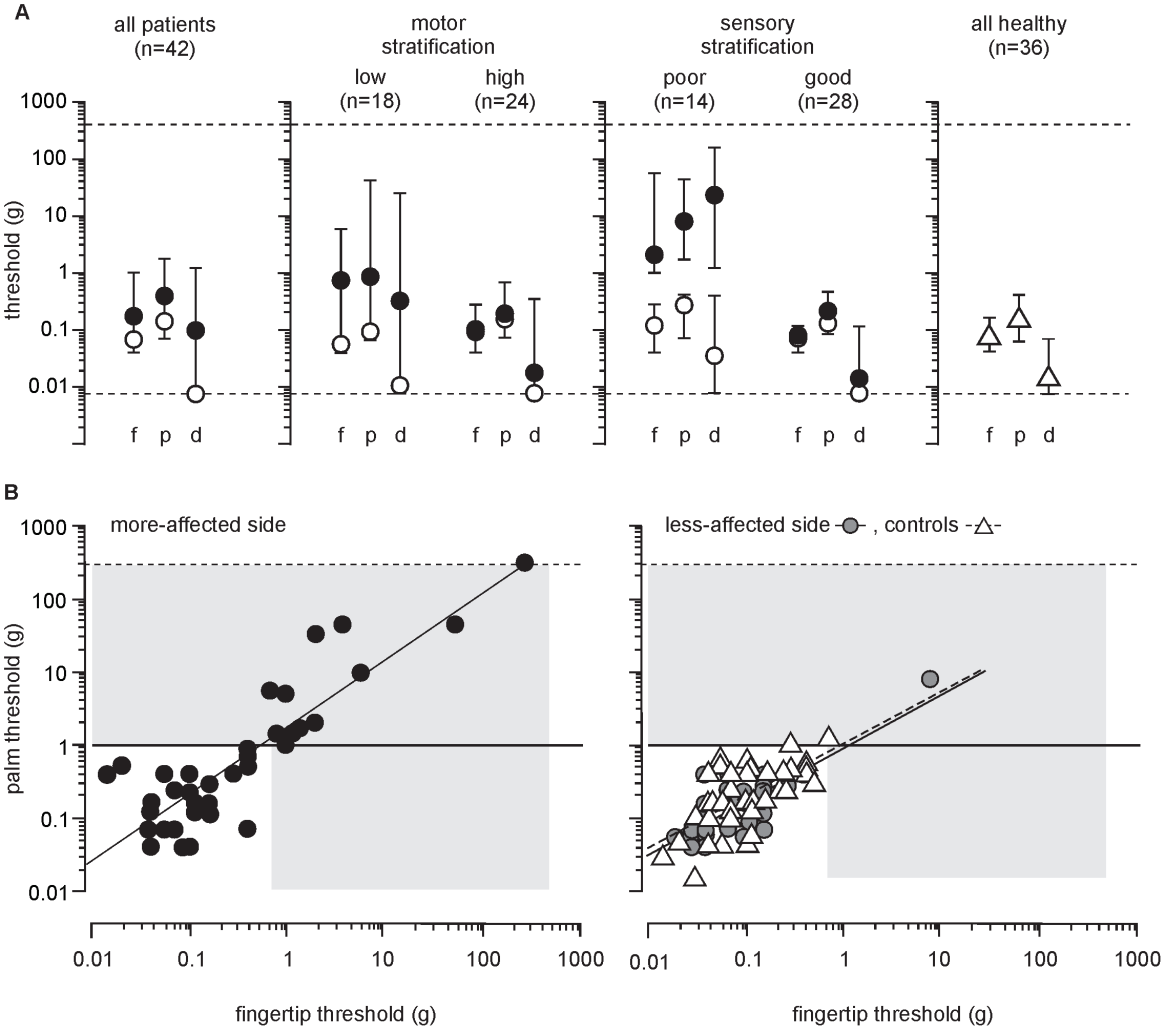
Cutaneous sensation after stroke and in age- and sex-matched healthy subjects A) Cutaneous sensation according to classification. Higher numbers represent poorer sensation (higher thresholds), data presented as median and interquartile range on a logarithmic scale (log_10_). Dashed lines indicate minimum and maximum monofilament size (0.008–300 g). Open circles: less-affected side; filled circles: more-affected side. Open triangles: healthy subjects, combined hands. Regions: f: fingertip; p: palm; and d: dorsum. ***B) Correlation between fingertip and palm thresholds.*** The zone of impaired sensation is indicated by grey shading (fingertip 0.6 g, palm 1.0 g, see text for details). The left hand panel illustrates a significant positive correlation between the fingertips and palm thresholds for the more-affected side (r_s_(40) = 0.79, p<0.001); the right hand panel illustrates the correlations for the less-affected side (r_s_(40) = 0.58, p<0.001), and healthy subjects (r_s_(34) = 0.64, p<0.001). r_s_ = correlation co-efficient (degrees of freedom).

The median data for both hands of stroke patients fell within the healthy range (Fig. 2A). There was an effect for side tested for all regions: fingertip (H(2) = 10.6, p = 0.005); palm (H(2) = 10.7, p = 0.005); and dorsum (H(2) = 13.9, p<0.001) with more-affected side thresholds higher at all regions than both the less-affected side and healthy subjects (p<0.05). There was no difference between the less-affected side and healthy subjects for any region. Significant positive correlations were found between fingertip and palm thresholds for both sides of patients (Fig. 2B). There was no correlation between patient sensory thresholds and either time-since-stroke or age at any region.

#### Motor-classified patient groups

After classification by motor-function the median fingertip and palm thresholds, but not dorsum, fell within the zone of impairment. An effect was found for motor-function level for all regions: fingertip (H(4) = 18.2, p = 0.001), palm (H(4) = 15.4, p = 0.004), and dorsum (H(4) = 20.1, p<0.001, Fig. 2A). Patients with low motor-function had higher sensory thresholds on the more-affected side in all regions compared to: i) their less-affected side (p<0.05); ii) healthy subjects (p<0.05); and iii) the less-affected side of patients with high motor-function (p<0.05); but not the more-affected side of patients with high motor-function.

#### Sensory-classified patient groups

After classification by cutaneous sensory-function the extent of sensory impairment became evident. All thresholds on the more-affected side of those with poor sensation fell in the zone of impairment and unlike healthy subjects, thresholds were highest on the dorsum. There was an effect for sensory-function level for all regions: fingertip (H(4) = 39.3, p<0.001), palm (H(4) = 39.8, p<0.001) and dorsum (H(4) = 39.6, p<0.001). Thresholds for patients with poor sensation were higher on the more-affected side at all regions compared to: i) their less affected side; ii) both sides of patients with good sensation; and, iii) healthy subjects (p<0.05, Fig. 2A). There were no differences between thresholds on the less-affected side of patients with poor sensation, both hands of patients with good sensation, and healthy subjects (Fig. 2A). There was no correlation between sensory thresholds in patients with poor sensation and time-since-stroke or age, at any region.

#### Healthy subjects

Two healthy subjects had dominant hand thresholds in the zone of impairment for 1 and 2 regions (aged 61 and 72, respectively). There was a significant effect for site of threshold (F(2,210) = 26.1, p<0.001, Fig. 2A), but no interaction with side-tested. *Post hoc* analyses showed cutaneous thresholds (mean±SE) were lower on the dorsum, 0.05±0.02 g compared to the fingertip, 0.13±0.02 g (p = 0.003) and palm 0.24±0.02 g (p<0.001). Palm thresholds were positively correlated with, but higher than fingertip thresholds (p<0.001, Fig. 2B).

### Relationship between cutaneous sensation and motor-function

WMFT and FMA data (Table 1) highlight the difference in motor-function between the high and low motor-function groups (H(1) = 30.2, p<0.001; F(1,40) = 162.3, p<0.001, respectively). Patients with poor cutaneous sensation had lower FMA scores (H(1) = 6.23, p = 0.013) and a non-significant trend to slower WMFT times (H(1) = 3.4, p = 0.064). Significant correlations were found between cutaneous sensation at all three regions and for both tests of motor-function (Table 2). Analyses of the MALQOM scores in a subset of patients (n = 30) showed a trend towards a correlation with sensory thresholds in the fingertips (correlation co-efficient r_s_ = −0.342, p = 0.06), but no relationship to thresholds on the hand or dorsum.

**Table 2:** Correlation between cutaneous thresholds and motor-function tests.

Test	Fingertip thresholds	Palm thresholds	Dorsum thresholds
WMFT time	r_s_(40) = 0.37 p = 0.018	r_s_ (40) = 0.33 p = 0.035	r_s_ (40) = 0.31 p = 0.045
FMA score	r_s_ (40) = −0.45 p = 0.003	r_s_ (40) = −0.40 p = 0.009	r_s_ (40) = −0.35 p = 0.025

Correlations were performed separately for each test of motor-function. r_s_ = correlation co-efficient (degrees of freedom).

## Discussion

This study was the first to: i) quantify sensory loss in chronic stroke patients, ii) examine multiple sites on the hand, and iii) correlate these changes with different levels of motor-function after stroke. Cutaneous sensation was significantly impaired on the more-affected side for 33% of patients compared to healthy subjects (p<0.05). Our key finding is that the presence and *magnitude* of sensory changes on the more-affected side will not be apparent if data are pooled. As expected, significant changes in sensation were only apparent when patients were classified with good or poor sensation. In this study, sensation on the more-affected hand fell within healthy limits when data were pooled, and showed only minor impairments at the fingertip and palm if a traditional motor-function classification was used. Once poor sensation was identified for 36% of patients, the level of impairment was at least an order of magnitude greater than when patient data were pooled. The majority of patients with low motor-function had worse cutaneous sensation than those with high motor-function, but impaired cutaneous sensation was not limited to those with poor motor-function. Cutaneous sensory changes were unilateral except for a single patient, and regardless of classification, the less-affected side was unimpaired. Our results highlight the persistence of unilateral sensory deficits after stroke, and stress the importance of appropriate classification when investigating the magnitude of sensory loss post-stroke.

### Cutaneous sensory impairment in the hand post-stroke

Sensory impairments have not been well characterized in chronic stroke patients. The *prevalence* of impaired cutaneous sensation in this study was lower than previously reported for patients >6 months post-stroke, in which sensation was categorized as ‘present’, ‘absent’ or ‘impaired’ [18]. Previous reports of greater bilateral impairments in acute and subacute patients [7], [8], [11] were not supported in this study and this may reflect our use of monofilaments, or improvements in contralesional cortical connectivity due to the resolution of diaschisis in the chronic stage post-stroke [50]. The *magnitude* of sensory deficits has not been quantified in community-dwelling stroke patients previously. Most quantitative reports examined the acute or rehabilitation phase [9], [11] and generally pool patients with good and poor sensation into a single cohort. As illustrated in this study, pooling patients with good and poor sensation will underestimate the magnitude of sensory impairments that potentially contribute to increased motor disability.

The larger finger representation in the somatosensory cortex was thought to predispose the digits to greater post-stroke sensory impairments [51]. In this study, sensory thresholds were lowest in the fingertips for the stroke patients, although palm and fingertip thresholds were positively correlated (Fig. 2B). In contrast, thresholds were highest on the dorsum, the site of lowest thresholds in healthy ageing [31]. The relationship between cutaneous sensation on the more-affected fingertip and palm was stronger than for healthy subjects and the less-affected side. Our results suggest impaired sensory thresholds in the hand can predict thresholds in the fingertips and vice versa, but further work is needed to clarify this relationship. These findings suggest a more systemic, presumably central impairment after stroke most likely associated with lesion size and location. Alternatively, the higher density of cutaneous receptors in the fingertip [52], strong coupling between the somatosensory and primary motor cortices, or greater redundancy in cortical processing may provide greater resilience to fingertip sensation.

### Cutaneous sensation and motor-function after stroke

The loss of large diameter afferent signals affects motor control particularly in the absence of vision [1], [53], [54]. Although the relationship between reduced fingertips sensation and impaired motor control are inconclusive or contradictory [23], [24], [55], monofilament thresholds ≥0.6 g are suggested to reflect diminished protective sensation that limits fine motor control of the hand [42]. We previously demonstrated that the age-related changes in cutaneous sensation and motor control were not strongly interdependent [33], but this does not imply independence of motor-function and sensory feedback. Our hypotheses were confirmed in that cutaneous sensation on the more-affected side was worse in patients with low motor-function. This may signify a larger lesion impacting the adjacent motor and sensory cortices. Alternatively, increased hemiparesis and subsequently reduced peripheral afferent signaling in these patients may have created maladaptive changes in central sensorimotor integration [28], with outcomes similar to that seen with learned non-use in motor control. Although we saw no correlation between sensation and time-since-stroke or use of the hand, these findings need to be confirmed in a larger cohort.

Sensation on the palm and fingertips was associated with motor-function, but not strongly. Importantly, poor cutaneous sensation was not confined to those with low motor-function but also included five patients with high motor-function. This finding may suggest that sensory impairments contributes little to motor impairments in people with severe motor dysfunction, or that a larger sample of patients with low motor-function is required. Alternatively this relationship may be stronger if more sensitive assessments of motor-function are developed for patients with low function, those more likely to have impaired sensation.

### Study Limitations

The incidence and nature of sensory loss in this study may have been *underestimated* by the relatively small cohort and the heterogeneity of patients recruited. Regardless, it is the first quantification of sensory loss in community-dwelling stroke patients. We acknowledge our findings may be directly related to the size and location of the cortical lesions but scan information was not available for all patients. We did not see any effect for age, presumably due to the similarity of ages across the cohort. Age may be a greater contributor to sensory thresholds in larger studies that incorporate broader age ranges or younger subjects. We also acknowledge there are limitations with the use of the motor assessments used in this study. The FMA in particular comprises ordinal scores which may result in an underestimation of the relationship between sensory thresholds and motor-function. Finally our results may have been influenced by pre-morbid sensory impairments that were not identified during patient screening. We saw no evidence indicative of these conditions, and do not believe it was a significant contributor to our findings.

### Clinical Implications

Our results demonstrate that the magnitude of sensory impairments is not evident if sensory data are pooled for analysis. Identifying both the incidence and magnitude of sensory impairments is important to understand the origin of functional limitations after stroke, to avoid bias and set appropriate inclusion criteria in clinical studies, and to reveal improvement in clinical rehabilitation trials. Given the importance of cutaneous sensation to motor control (see Introduction), impaired cutaneous sensation provides a potential avenue for therapeutic intervention that may contribute to improved motor-function through the restoration of more effective sensorimotor integration [55]. We acknowledge that classification by sensory level has less application in clinical practice where patients are assessed and treated on an individual basis. Regardless, the persistence of significant sensory impairments in one-third of these community-dwelling stroke patients reinforces the importance of developing sensory re-training tools suitable for all levels of function.

Testing cutaneous sensation with monofilaments is time-consuming and requires participant concentration to elicit meaningful perceptual judgments. However, traditional clinical testing may miss subtle but clinically important changes in sensory function, underestimating sensory impairment. We recently showed that monofilaments remained one of the most sensitive measures of age-related change in cutaneous sensation of healthy populations [31], and here confirm they can be used to quantify sensation after stroke with different levels of motor-function. This test required minimal verbal responses and can be used regardless of expressive language dysfunction. In this study impaired cutaneous sensation was classified as values ≥3 SD from the mean for healthy subjects based on monofilament fingertip thresholds in healthy subjects [42]. Age-appropriate thresholds for impaired sensation on the palm and dorsum do not exist, so we extrapolated from the fingertip criteria to reflect the higher palm thresholds and lower dorsum thresholds [56]. The clinical relevance of the thresholds for impaired sensation established here requires confirmation in larger populations.

### Conclusions

This is the first study to quantify the magnitude of cutaneous sensory impairments for community-dwelling stroke patients. Significantly impaired sensation on the more-affected side was not evident when data were pooled. Cutaneous sensation was worse with low motor-function and further studies are required to determine to what extent specific sensory training can improve dexterous motor-function necessary for independence in activities of daily living. This study demonstrates the importance of appropriately examining data to avoid masking potentially important impairments in sensation.
